# Utility-free heuristic models of two-option choice can mimic predictions of utility-stage models under many conditions

**DOI:** 10.3389/fnins.2015.00105

**Published:** 2015-04-09

**Authors:** Steven T. Piantadosi, Benjamin Y. Hayden

**Affiliations:** ^1^Department of Brain and Cognitive Sciences, University of RochesterRochester, NY, USA; ^2^Center for Visual Science, University of RochesterRochester, NY, USA

**Keywords:** decision making, value comparison, heuristics, dimensional prioritization, value correlate, utility

## Abstract

Economists often model choices as if decision-makers assign each option a scalar value variable, known as utility, and then select the option with the highest utility. It remains unclear whether *as-if* utility models describe real mental and neural steps in choice. Although choices alone cannot prove the existence of a utility stage, utility transformations are often taken to provide the most parsimonious or psychologically plausible explanation for choice data. Here, we show that it is possible to mathematically transform a large set of common utility-stage two-option choice models (specifically ones in which dimensions are can be decomposed into additive functions) into a heuristic model (specifically, a dimensional prioritization heuristic) that has no utility computation stage. We then show that under a range of plausible assumptions, both classes of model predict similar neural responses. These results highlight the difficulties in using neuroeconomic data to infer the existence of a value stage in choice.

## Introduction

How do our brains choose between two differently valued options? One straightforward strategy would be to assign each option a scalar value variable (called *utility*) and then choose the option with the highest utility (von Neumann and Morgenstern, 1944). Utility has been a core concept in economic theory since the birth of economics in the eighteenth century (Bernoulli, 1738; Samuelson, 1938; Houthakker, 1950/2002). Decision-makers who adhere to basic principles of rationality can be shown to behave as if they compute and compare utilities (Savage, 1954). But do we *actually* compute and compare utilities? Economists have traditionally refrained from speculating (Samuelson, 1953). Indeed, choice behavior by itself cannot confirm the existence of a value stage; for this reason, some scholars have turned to neuroeconomics.

Many popular neuroeconomic models of choice resemble the *two-stage model* (Padoa-Schioppa, 2011). At the core of this model, options are first evaluated, meaning that a single scalar quantity, known as utility, is computed and assigned to that option. Importantly, the same scale is used for all options being compared. Then their utilities (and only utilities) are compared. Thus, for the purposes of this paper, we define utility as a single scale that can be used to compare any set of values, and that has a discrete value for each option. In standard models, evaluation is discrete, in the sense that each option is given its own specific value (even if this value depends on the other offers available). The evaluation stage creates a single scalar value variable (*utility*). The utility variable includes all factors that influence choice, including, for example, delays, risk, gustatory value, effort costs, and more esoteric factors like the value of the information the option provides (e.g., Padoa-Schioppa and Assad, 2006; Rudebeck et al., 2006; Blanchard et al., 2015).

The question of utility's reality has come to prominence because of neuroscientists' suggestion that utility has a neural instantiation (Montague and Berns, 2002; Rangel et al., 2008; Padoa-Schioppa, 2011; Levy and Glimcher, 2012). Brain activity often correlates closely with utility; this correlation is often thought to provide evidence for the neural reality of an evaluation stage (e.g., Knutson et al., 2001; Kim et al., 2008; Chib et al., 2009; Kennerley et al., 2009; Boorman et al., 2013; Blanchard and Hayden, 2014). Indeed, brain recordings directly support the idea that we make choices by computing a utility for each option and then choosing the option with the highest utility (Padoa-Schioppa and Assad, 2006; Wunderlich et al., 2010; Hunt et al., 2012; Strait et al., 2014, in press). These data suggest that utility is a critical step in the algorithm the brain uses to implement choices, and endorse the reality of economists' heretofore hypothetical models.

Despite this evidence, it is still not clear that we make choices by computing and comparing utilities. First, neural correlates of value are notoriously difficult to attribute to value *per se*; value often correlates with attention, arousal, salience, and other factors (Maunsell, 2004; Heilbronner et al., 2011; Schoenbaum et al., 2011; Leathers and Olson, 2012; O'Doherty, 2014). Second, a great deal of research in behavioral economics demonstrates the surprising power of utility-free choice models to account for choices (Brandstätter et al., 2006; Hogarth and Karelaia, 2007; Stewart et al., 2014; see Vlaev et al., 2011 for a detailed review). Because these models are in many cases process models, they make direct predictions about the mental steps in choice. Utility-free models suggest the possibility that the concept of utility may be a convenient way of thinking about an emergent process, but that seeming representations of utility are just coincidental correlates of utility. Utility-free models are thus *eliminative*, meaning that they open up the possibility that utility can be classified with vitalism or the four elements theory of matter as folk theories that do have a direct correspondence to underlying reality (Churchland, 1981).

Computations that avoid computation utility often involve heuristics—simple rules that generate choices. The results of such rules or sets of rules can produce quite good choices in the aggregate. Some utility-free models include decision-by-sampling theory, fuzzy trace theory, query theory, elimination by aspects, and dimensional prioritization (Tversky, 1969, 1972; Stewart et al., 2006, 2014; Johnson et al., 2007; Reyna, 2009). In many circumstances, these strategies can predict behavior as well as or better than those of comparable utility-stage models (Kahneman et al., 1982; Payne et al., 1993; Gigerenzer and Goldstein, 1996; Brandstätter et al., 2006). A few recent studies demonstrate the neural viability of heuristic approaches as well (Fellows, 2006; Venkatraman et al., 2009a). Heuristics provide an appealing process model because they are quite flexible (Payne, 1976; Kahneman et al., 1982; Brandstätter et al., 2006) and because they may be less mentally effortful than evaluation and comparison (Tversky, 1969; Martignon and Hoffrage, 2002; Hogarth and Karelaia, 2007; Shah and Oppenheimer, 2008).

Here we focus on binary two-attribute choices, which are perhaps the most well studied types of problems in decision-making and include many risky choices, intertemporal choices, purchasing, self-control, foraging decisions, many game theoretical problems, and classic social choice problems such as the ultimatum game. We show that such choices can be modeled with a prioritization heuristic (Tversky, 1969; Payne, 1976; Russo and Dosher, 1983; Hsee et al., 1999; Katsikopoilos and Gigerenzer, 2008; Scholten and Read, 2010). This heuristic involves identifying the various dimensions along which choice options vary, selecting the dimension with greatest variance, and then choosing the option that dominates along the prioritized dimension. We then show that this approach applies to a broad class of neuroeconomically interesting decisions.

Choices made using this heuristic are theoretically interesting because they do not have an evaluation step and thus avoid the computation of utility. Evaluating a single dimension for priority does not require utility computation because it ignores information on all other dimensions. Similarly, comparing dimensions does not require utility computation because it occurs in an abstract unitless space that depends on properties of multiple options but is blind to the identities of the options. In other words, dimensional comparison gives no information about which option will be chosen. Finally, the choice stage does not involve a utility computation because it only occurs within a given dimension. Earlier work by Tversky showed that under some conditions, it is possible to reframe a utility model (which he called a horizontal model) to a utility-free one (which he called a vertical model, Tversky, 1969).

We extend Tversky's results by showing that it is possible to mathematically transform many binary choice models into dimensional prioritization heuristic form that has no utility computation stage. We also show that any utility function with any finite number of attributes that can be decomposed into additive functions of its attributes has a psychologically plausible utility-free dimensional prioritization equivalent that predicts the same choices. Our results are restricted to two option choices with arbitrarily many variables subject to arbitrary transformations, as long as those transformations are decomposable into additive functions of dimensions. These conditions include a large number of well-known choice contexts, including hyperbolic and exponential discounting, Bernoullian risk attitudes, cost/benefit decisions, and parts of Prospect Theory.

We also show that neural correlates of value difference cannot be taken to exclusively imply a value comparison. Indeed, across multiple trials, this variable is closely correlated with value of both offered and chosen options (under reasonable assumptions). For this reason, neural value correlates in such tasks do not necessarily imply evaluation processes. Thus, while neural activity measured in such tasks can support the involvement of brain cells in economic choice, it is necessarily ambiguous about whether there is a specific evaluation stage in that choice (O'Doherty, 2014).

## Results

### The dimensional prioritization heuristic

Although real-world decision makers often have complex utility functions, we will first consider the simplest case: a hypothetical decision-maker faced with a chose between two gambles who has a strictly linear utility function over the range of possible values and no distortions in its treatment of the probability curve. The utility this decision maker assigns to each gamble is identical to the gamble's mathematical expected value. Such a decision maker might correspond, for example, to the policy used by an investment bank or an insurance company to evaluate simple loans or policies.

Imagine this decision-maker is faced with a gamble defined by a probability *p* of winning a reward *R* and a probability (*1–p*) of winning a reward of zero. The decision-maker's subjective value of this option is simply its mathematical expected value, *EV* = *p* · *R*. Our decision-maker will have a utility function that matches EV, and thus *U* = *EV* = *p* · *R*. To choose between two gambles [*p*_1_ *R*_1_] and [*p*_2_ *R*_2_], the decision-maker can make the utility-maximizing choice by obeying the following steps:

**Algorithm 1:** **(Utility algorithm)**.

1.	U_1_ = p_1_ · R_1_
2.	U_2_ = p_2_ · R_2_
3.	Select the option with the greater utility

This algorithm computes a utility variable (and in fact does so twice, once for each option). Stages 1 and 2 can be considered **evaluation stages**, since they assign a specific value to specific options. The value they compute is scalar and allows for comparison between dissimilar options; it is thus a common currency model of choice (Montague and Berns, 2002; Padoa-Schioppa and Assad, 2006; Padoa-Schioppa, 2011).

The central question we asked is whether it is possible to generate an algorithm that lacks a utility stage but makes all the same choices as Algorithm 1. The reason an alternative algorithm that makes the same choices but has no utility stage is scientifically interesting is because, if such an algorithm exists, it would be impossible to ascertain which algorithm was used to generate the choices, even with an arbitrarily large dataset.

The algorithm we discuss here is a ***dimensional prioritization heuristic***. In general, a dimensional prioritization heuristic identifies one dimension along which options vary and then selects the option with the preferred value along that dimension (Brandstätter et al., 2006). A dimensional prioritization heuristic is utility-free because dimensions are considered separately at all points in the task. As a trivial example, a hypothetical lottery ticket buyer may simply ignore all information about probabilities and focus on the amount winnable, and choose the contest with the highest jackpot even in cases when it has a lower expected value. This gambler's dimensional prioritization heuristic is clearly costly. However, a gambler who carefully selects which attribute to attend to may make wiser choices.

The two gambles [*p*_1_ *R*_1_] and [*p*_2_ *R*_2_] differ along two dimensions (sometimes called attributes), probability (*p*) and reward amount (*R*). How does the decision-maker choose which dimension to prioritize in its choices? One possibility is to choose the dimension with more variance. This is a plausible strategy because that dimension will likely convey more information about the options. As a measure of variance, we use the statistical concept of *relative difference*. The relative difference of the probability dimensions is:

(1) $$RD_{prob}=\left|\frac{p_{1}-p_{2}}{mean(p_{1},p_{2})}\right|$$

The relative difference of the reward dimension as:

(2) $$RD_{rwd}=\left|\frac{R_{1}-R_{2}}{mean(R_{1},R_{2})}\right|$$

Then, a decision-maker could implement the following algorithm:

**Algorithm 2:** **(Utility-free dimensional prioritization algorithm)**.

1.	Calculate RD_prob_
2.	Calculate RD_rwd_
3.	Select the dimension with the larger RD value
4.	Select the option with the larger value along the selected dimension

This heuristic does not involve computation of utility at any point. The two RD variables (Steps 1 and 2) involve only a subset of the aspects of both options, and thus do not indicate the overall value of either. Both steps compute a value along a common scale, but their common scale is a dimension-free abstract scale, not a utility scale (or even, more generally, a reward value scale), so step 3 does involves evaluation of dimensions but not options. Likewise, the selection step (step 4) does not involve utility because it is limited to a single dimension.

### Proof that Algorithms 1 and 2 are mathematically equivalent

Here we show that Algorithm 1 and Algorithm 2 are mathematically equivalent, and thus produce the same choices. Assuming two gambles defined as [*p*_1_ *R*_1_] and [*p*_2_ *R*_2_]. For convenience, let us define two new terms *R* and *r* such that *R*_1_ = *R* + *r* and *R*_2_ = *R* – *r*. We will also assume terms *P* and *p* such that *P*_1_ = *P* + *p* and *P*_2_ = *P* – *p*. *R* is thus the average value of reward for the set of options and *P* is the average value of probability.

By Algorithm 1, option 1 is preferred if U_1_ > U_2_. Thus, option 1 is preferred if:

$$p_{1}\cdot R_{1}>p2\cdot R_{2}$$

Given our new terms *R*, *r*, *P*, and *p*, we can rewrite this inequality as:

$$\begin{array}{l} \left(P+p)\cdot (R+r)>(P-p)\cdot (R-r\right)\\ P\cdot R+p\cdot R+r\cdot P+p\cdot r>P\cdot R-p\cdot R-r\cdot R+p\cdot r\\ p\cdot R+r\cdot P>-p\cdot R-r\cdot P\\ \frac{2\cdot p}{P}>\frac{-2\cdot r}{R} \end{array}$$

Note that at this point, the comparison is made entirely within dimensions. No utility is computed. No common currency is used. The term *2p/P* is what is known in statistics as a relative difference between probabilities.

Critically, the best choice will depend on the sign of *p* and *r*. When *p* is positive and *r* is negative (i.e., option 1 has higher probability but lower reward), option 1 is better only if probability has a greater relative difference between the options than reward. Conversely, when *p* is negative but *r* is positive (i.e., option 1 has a lower probability but higher reward), option 1 is better only if reward has a greater relative difference than probability. In other words, this equation can be summarized with a very simple heuristic: look at the dimension (probability or reward) that has a larger relative difference between the two choices. Choose the winner based on which choice wins along that dimension alone which is equivalent to Algorithm 2. Algorithm 2 thus provably gives the same result as Algorithm 1.

### This relative difference heuristic works in many conditions

As presented so far, this model only applies to one specific decision model, the normative risk-neutral decision-maker. However, the general approach can easily be extended to choice models in which each dimension is arbitrarily deformed as long as the deformation function is a linearly separable from the other dimensions. For example, a popular model of risky choice, based on ideas originally proposed by Bernoulli, posits that the subjective value of winning is a monotonic but non-linear function of its nominal value (e.g., Yamada et al., 2013). In practice, the deformed subjective value function is often an exponential but does not have to be; it may more generally be some function *f*(*R*).

(Note that in practice this value function, before probability weighting, is sometimes called a utility function, and the decision variable is called the expected utility function. This terminology is confusing for present purposes because in that parlance the word utility does not refer to the decisional utility of the gamble, but to an earlier computational stage. For clarity therefore we will call it the subjective value of the reward and reserve the term utility for the final of the decisional value of the offer).

In the case of a general *subjective value function* for reward *f*(*R*), our dimensional prioritzation heuristic (Algorithm 2 above) can be made to make the same choices as the algorithm by replacing *R*_1_ and *R*_2_ with *f*(*R*_1_) and *f*(*R*_2_).

(3) $$RD_{prob}=\left|\frac{p_{1}-p_{2}}{mean(p_{1},p_{2})}\right|$$

(4) $$RD_{rwd}=\left|\frac{f(R_{1})-f(R_{2})}{mean(f(R_{1}),f(R_{2}))}\right|$$

Using these inputs, Algorithm 2 is still mathematically equivalent to Algorithm 1, and must predict the same choices. As long as *f*(*R*_1_) and *f*(*R*_2_) are not functions of *p*_1_ or *p*_2_, then Algorithm 2 still does not involve anything resembling a utility stage.

It is worth emphasizing at this point that our approach and our goals diverge from those of most heuristic approaches. The goal of a typical heuristic study is to identify extremely simple rules that can approximate formal rules under a broad range of conditions with a minimum of assumptions and fit parameters. Our goal instead is to demonstrate the existence of a psychologically plausible utility-free heuristic that has no utility computation that perfectly mimics utility-stage models. Thus, we are quite willing to add in the non-linear variable transformations (the functions denoted by *f* above and by *g* below). While these violate the spirit of minimalism that is common in heuristics, they are psychologically plausible.

Another choice model, prospect theory, involves both a Bernoulli-type non-linear transformation of value and a transformation of probability into decision weights (Kahneman and Tversky, 1979). (Prospect theory also involves an editing stage that we ignore here). This transformation is generally assumed to be monotonic (although it does not have to be). As long as it is not a function of reward, it is simple to compute new relative difference values that can be used with Algorithm 2. Assuming that *g*(*p*) is a reweighting of the probability curve:

(5) $$RD_{prob}=\left|\frac{g(p_{1})-g(p_{2})}{mean(g(p_{1}),g(p_{2}))}\right|$$

(6) $$RD_{rwd}=\left|\frac{f(R_{1})-f(R_{2})}{mean(f(R_{1}),f(R_{2}))}\right|$$

Thus, this stage of prospect theory has a utility-free equivalent. Moreover, there is nothing special about the fact that these choices involve risk. For example, in a well-known study, subjects choose between two amounts of juice that differ in flavor and quantity (Padoa-Schioppa and Assad, 2006). In another well-known study, subjects choose between options that differ along gamble amount and information value (Bromberg-Martin and Hikosaka, 2009). It is plausible to assume that in these studies, the utility of each option may be a product of its scalar values along the two dimensions. If so, it is straightforward to create a utility-free algorithm that makes the same choices as the choice model using the same principles.

Going even further afield, these results are not restricted to choice models in which utilities are defined as products of scalars; they also apply to quotients (i.e., ratios). In many decision contexts preferences are well described as the outcome of a comparison of benefit/cost ratios. One well-known example comes from decisions involving a tradeoff between reward and effort (Rudebeck et al., 2006; Walton et al., 2007). Another well-known example from foraging theory comes from the diet selection problem with simultaneous encounter; the value of each option is given by the ratio of reward/delay (Blanchard and Hayden, 2014). In this case, the Algorithm 2 can be used by replacing delay with its reciprocal:

(7) $$RD_{delay}=\left|\frac{D_{1}^{-1}-D_{2}^{-1}}{mean(D_{1}^{-1},D_{2}^{-1})}\right|$$

(8) $$RD_{rwd}=\left|\frac{f(R_{1})-f(R_{2})}{mean(f(R_{1}),f(R_{2}))}\right|$$

This case extends to slightly more complex quotients. One of the most well-known of these is the hyperbolic discounting equation:

(9) $$U=\frac{R}{1+k\cdot D}$$

In this equation, *k* is a constant, *D* is the delay until the option is given, and *R* is the original value. Despite its seeming complexity, this equation is fundamentally a ratio of reward to delay:

(10) U=R·Δ

Where Δ is simply defined as 1/(1 + k · D). This term Δ may look artificial but it has an intuitive psychological definition: it is an impulsivity-weighted measure of “soonness.” Having defined this *soonness* term, a relative difference can be straightforwardly computed:

(11) $$RD_{delay}=\left|\frac{\Delta_{1}-\Delta_{2}}{mean(\Delta_{1},\Delta_{2})}\right|$$

(12) $$RD_{rwd}=\left|\frac{R_{1}-R_{2}}{mean(R_{1},R_{2})}\right|$$

### Binary choices with more than two attributes

Above, we considered the most well-known case, decisions in which the two options vary along two dimensions. We next consider the case of two-alternative choice with options that vary along a finite number of discrete dimensions. The general form of a utility function for one option will be *U*(*x*), where *x* refers to the option and U refers to the utility function. For convenience, we will label each of the dimensions with a subscript. So the utility is *U*(*x*_1_, *x*_2_, *x*_3_, …, *x*_*n*_), and it can be written in the form of

(13) $$U\left(x_{1},x_{2},x_{3},...,x_{n}\right)=g\left(\sum_{i=1}^{n}f_{i}\left(x_{i}\right)\right).$$

We restrict ourselves here to the case where g is a monotonically increasing function: *R* → *R* and arbitrary functions *f*_1_, *f*_2_, … *f*_*n*_ with *f*_*i*_: *R* → *R*. (These assumptions apply to almost all decision models with which we are familiar). The letter *g* here refers to some monotonic operator on the outcome of the summation step. Thus, for example, we may have a diminishing marginal utility of value, which would lead to a convex function *g*.

Within this framework, the comparison between options x and y can be written as:

(14) $$U(x_{1},x_{2},...,x_{n})\geq U(y_{1},y_{2,}...,y_{n}).$$

In other words, the decision can be made by comparing scalar utilities that are themselves functions of a vector of scalar *dimension variables*. These dimension variables may be quantities like the probability of winning a gamble, the delay until the reward is given, the amount of information an option offers, and the flavor of the juice offered.

By simple replacement, this problem can be solved if we can determine whether:

(15) $$g\left(\sum_{i=1}^{n}f_{i}(x_{i})\right)\geq g\left(\sum_{i=1}^{n}f_{i}(y_{i})\right).$$

Because we are assuming that *g* is monotonic, Equation 3 can only be true in the case that:

(16) $$\sum_{i=1}^{n}f_{i}(x_{i})\geq \sum_{i=1}^{n}f_{i}(y_{i})$$

which can easily be rearranged to the following:

(17) $$\sum_{i=1}^{n}\left(f_{i}(x_{i})-f_{i}(y_{i})\right)\geq 0.$$

This equation requires only comparison within each dimension rather than the computation of an overall utility for each choice. In other words, the algorithm performs a separate comparison for each dimension, sums the differences, and compares the sum of the differences to zero. This is a utility-free approach because comparisons are made across dimensions first and only the differences are carried onto the next step of the operation. At no point in the process is the utility of a single option a part of the calculation.

Note that these findings are anticipated in Tversky (1969), who discussed rearrangements like those converting Equation (16) to Equation (17). However, while he first identified this general principle, here we point out that the same framework can accommodate a monotonic transformation (*g*). This change is mathematically minor, but has important practical consequences as it allows reframing of multiplicative terms into the requisite (additive) sum through choosing g(x) = exp(x). This transfers a much broader class of problems into the framework Tversky identified.

### Working through the cases for some well-known examples

Although Equation (13) is a restricted form, the flexibility of the *f*_*i*_ term makes it very general a broad class of utility functions. Here we show how to generate linearly separable functions for some well-known choice models:

#### Utility is a mathematical expected value

(or, more generally, the product of two scalar variables)

U(p,r) =p·r

Let *g*(s) = e^s^ (i.e., exp(s)); f_1_(p) = log(p), f_2_(r) = log(r).

Then U(p, r) = g(f_1_(p) + f_2_(r)) = exp(log(p) + log(r)) = exp(log(p · r)) = p r.

This case is most intuitive for expected values, but applies in any conditions in which two variables are combined by multiplying to compute a utility.

#### Utility is the product of linear transformations of two scalar variables

U(p,r) = s(p)·t(r)

Let g(s) = exp(s), f_1_(p) = log(s(p)) and f_2_(r) = log(t(r)).

Then (following the same logic as above), U(p, r) = g(f_1_(s(p)) = f_2_(t(r))) = exp(log(s(p)) + log(t(r))) = exp(log(s(p) · t(r)) = s(p) · t(r).

#### Utility is a weighted hyperbolically temporally discounted reward

$$U(A,R,D)\;=\;A\cdot \frac{R}{1+k\cdot D}$$

Let g(s) = exp(s), f_1_(A) = log(A), f_2_(r) = log(r) and f_3_(D) = −log(1 + k · D).

#### Utility is a weighted exponentially temporally discounted reward

$$U(A,R,D)\;=\;A\cdot e^{-k\cdot D}$$

Let g(s) = exp(s); f_1_(D) = log(exp(k · D)) = kD.

### Implications for neural signals

These findings have implications for the interpretation of neural data. Neuroeconomic studies typically involve the simultaneous presentation of a pair of offers followed by choice and resolution of the choice (e.g., determining the outcome of a gamble). Because neural measures are inherently noisy, standard practice is to create large datasets by aggregating across trials.

The most important putative neural signature of evaluation processes is the utility signal itself. That is, this signature is a correlation between the utility of one of the two offers and neural activity, whether that be firing rate of a single neuron, BOLD activity of fMRI, or some other measure. However, much of our math also applies to neural correlates. Thus, consider a single offer consisting of two dimensions where utility is determined by the product of their values. The utility of that offer is correlated with both the values individually. Assuming offers are chosen in such a way as to span the space of utilities, averaging across many trials, the utility of the offer is correlated with the value of both individual dimensions. This remains true even if only one dimension is encoded on each trial.

To demonstrate this, we used a simulation to study the expected correlation between hypothetical neural signals representing utility and signal representing single dimension choice rule in Algorithm 2. We simulated a large number of presented choices with probabilities and rewards for two options uniformly distributed in [0,1]. For each, we computed the expected value difference, as well as a signal relevant to the dimensional prioritization algorithm: the difference in the dimension (probability or reward) that has the higher relative difference.

**Figure 1:**
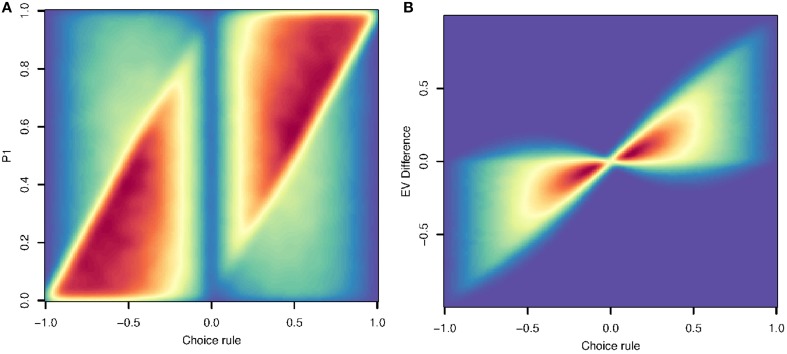
**(A)** This density plot shows the difference in the dimension chosen by the dimensional prioritization algorithm (x-axis) vs. the value of P1 (y-axis) in a simple simulation. The strong correlation (*R* = 0.45) indicates that a neural representation of the key comparison in step 4 of Algorithm 2 can *appear* erroneously to be representing the probability of choice 1, or other variables (see text). **(B)** The correlation between the difference in the dimension chosen by the dimensional prioritization algorithm (x-axis) and the difference in expected value (y-axis). The correlation (*R* = 0.83) here demonstrates that a neural representation of step 4 in Algorithm 2 can also appear erroneously to be representing the difference in expected value.

Figure 1A shows the probability of choice 1 as a function of the difference between choices in the dimension with the greater relative difference. These two are correlated at *R* = 0.45. This means that a neural signal thought to be correlated with *P*_1_ (at *R* ≤ 0.45) might actually reflect a representation that is part of the dimensional prioritization algorithm. Note that in our simulation *P* and *R* have analogous roles and distributions, so this correlation for *P*_1_ is also found for *P*_2_, *R*1, and *R*_2_.

Even more, the correlation between algorithmic components holds for utility as well. Figure 1B shows the correlation between the difference in the dimension with greater relative variance against the expected value difference. This correlation is even higher, *R* = 0.83, meaning again that neural signals that appear to be correlated with expected value might actually reflect representations of the dimensional prioritization algorithm. Given our math in preceding sections, similar qualitative results will necessarily hold for choices involving more factors.

In short, these figures show that neural correlates of utility in the aggregate may instead reflect encoding of one dimension. For this reason, single unit correlates of utility are confounded with a core variable in the heuristic algorithm. This applies just as well to aggregate measures of neural activity (BOLD signal for example), but because these methods average across neurons, they have an additional confound: individual neurons could consistently encode one variable but as long as they are intercalated within a voxel, the activity of the voxel will track the utility of the option, even as no neurons do.

## Discussion

We find that a broad range of binomial choice models that involve an explicit utility computation stage have a mathematical equivalent that does not involve computation of utilities. In other words, there exists at least one class of utility-free heuristics flexible enough that it can generate utility-free doppelgangers of utility models that make identical predictions. These alternatives involve evaluation and comparison of dimensions rather than evaluation and comparisons of options. These results apply to a large set of binomial decisions with arbitrarily many dimensions; the dimensions can be deformed although the dimensions must be decomposable into additive functions.

It is well established that choice behavior alone cannot confirm the existence of a utility stage in choice; however, we show that utility-free alternatives are available that are simple and psychologically plausible. We further show that neural correlates of utility that have been tabulated across multiple choices may equally reflect intermediate stages of the heuristic. Consequently neuroeconomic data providing clear correlates of utility computations can arise from utility-free heuristics. These results suggest that confirming the existence of a discrete evaluation stage in choice may be more difficult than is generally believed.

Many scholars have identified heuristics or other simple choice models, not limited to dimensional prioritization, that provide better descriptions of behavior than utility models (Tversky, 1969, 1972; Kahneman et al., 1982; Payne et al., 1993; Gigerenzer and Goldstein, 1996; Brandstätter et al., 2006; Stewart et al., 2006, 2014; Johnson et al., 2007; Hayden and Platt, 2009; Reyna, 2009; Venkatraman et al., 2009a; Pearson et al., 2010; Blanchard and Hayden, 2015). Our goals here are somewhat different: we are interested in whether it is possible to infer the existence of a utility stage from a combination of behavioral and neural data. We are agnostic about whether decision-makers actually employ the dimensional prioritization heuristic as long as it is plausible. Indeed, they probably don't: there is evidence that we select from a large number of possible strategies so that strategy varies with the situation (Payne et al., 1993; Gigerenzer and Selten, 2002; Heilbronner and Hayden, 2013). Nor do our results suggest that decision-makers favor utility-free decision strategies—although many others do (Tversky, 1969, 1972; Kahneman et al., 1982; Payne et al., 1993; Brandstätter et al., 2006; Stewart et al., 2006; Scheibehenne et al., 2007; Hayden and Platt, 2009; Scholten and Read, 2010; Vlaev et al., 2011; Blanchard et al., 2013; Strait and Hayden, 2013).

One limitation of our findings is that they do not deal with situations of perfect ambivalence, i.e., when two rewards are perfectly matched in subjective value. This is, of course, just as much of a problem for utility-based models. However, we believe that the problem is not particularly important in either case; adding a small dispersion term or even a modicum of stochastic variability to either model will eliminate this problem. Another major limitation of our results is that they do not apply to multi-option (more than two) choice. Indeed, a strict dimensional prioritization heuristic cannot handle three-option choices in which one option is closely dominated on both dimensions by the one of the other two options but strongly dominates on the other dimension. However, we suspect this weakness may be less serious than it first appears. In practice, humans are quite poor at multi-option choice and very often resort to utility-free heuristics to reduce their set of options before deliberation (Tversky, 1972; Payne et al., 1993). In any case, it remains an open question whether utility models for multi-option choices have a utility-free mathematical equivalent.

### Implications for neuroscience

In many studies of the neural basis of economic choice, a subject chooses between two options that differ along two or more dimensions and a best-fitting evaluation function is computed from the subject's behavior. (Common examples include risk functions and temporal discounting functions). If measures of brain activity correlate with the value predicted from the evaluation function, it is sometimes inferred that they are utility correlates and thus presumably utility representations—a signature of an evaluation stage. Our findings suggest that the existence of such utility correlates may arise artifactually from utility-free heuristic processes.

Specifically, our model generates a decision variable that is simply a scalar representation of one of the task dimensions, not an integrated utility variable. Of course, the identity of this variable differs depending on the parameters of the options, and then, in the aggregate, has the same statistics that a utility variable would—even though it is different from utility on each trial. Given the equivalence of the heuristic and utility models, this difference is wholly irrelevant for predicting behavior. However, it means that any neural variable that covaries with utility on average may instead covary with the output variable of the heuristic on individual trials and with utility only on average. Thus, we have identified a potential confound to neural correlates of utility. This confound is in addition to other well-known confounds like salience, attention, and arousal (Maunsell, 2004; Heilbronner et al., 2011; Schoenbaum et al., 2011; Leathers and Olson, 2012; O'Doherty, 2014).

Our results do not imply that it is impossible to distinguish utility-based choices from dimensional prioritization heuristics. Data from carefully designed experiments, even data as simple as reaction times and eye movements, can shed light into the mechanisms of choice (Krajbich et al., 2010; Kacelnik et al., 2011; Pais et al., 2013). Of course, direct measures of brain activity are likely to be even more helpful. For example, we conjecture that the heuristic algorithm, but not the utility algorithm, would elicit neural control signals that are categorically different depending on the prioritized dimension, and also adjudication processes that are strongest when dimensions are close in variance. Future studies will be needed to test these hypotheses (for work in this direction, see Venkatraman et al., 2009a,b).

Finally, we speculate that utility may not be reified in the brain at all, but may be an emergent property of the algorithm that produces choices. It may be a scientifically useful concept to describe behavior in terms of utility, just as minimization equations can model a soap film even if the film itself lacks any explicit representations of the function it minimizes. To use the terminology of Marr, the computation stage should be distinguished from the algorithmic stage (Marr, 1982). To use the terminology of the philosopher Paul Churchland, a dimensional prioritization heuristic *eliminates* the concept of reward from reward-based choice (Churchland, 1981). Elimination in this sense refers to the philosophical position that basic folk psychology concepts like beliefs, intentions, and desires do not correspond to coherent neural processes, but are instead emergent properties of neural architecture and function. While the present results do not provide evidence for or against the position that the psychological concept of utility should be eliminated, they suggest it may be possible to do so.

### Conflict of interest statement

The authors declare that the research was conducted in the absence of any commercial or financial relationships that could be construed as a potential conflict of interest.
